# Seasonality in pulmonary tuberculosis among migrant workers entering Kuwait

**DOI:** 10.1186/1471-2334-8-3

**Published:** 2008-01-07

**Authors:** Saeed Akhtar, Hameed GHH Mohammad

**Affiliations:** 1 Department of Community Medicine and Behavioural Sciences, Faculty of Medicine, Kuwait University PO Box 24923, Safat 13110, Kuwait; 2 Ports and Borders Health Division, Ministry of Health, PO Box 32830, Rumaithiya 25410, Kuwait

## Abstract

**Background:**

There is paucity of data on seasonal variation in pulmonary tuberculosis (TB) in developing countries contrary to recognized seasonality in the TB notification in western societies. This study examined the seasonal pattern in TB diagnosis among migrant workers from developing countries entering Kuwait.

**Methods:**

Monthly aggregates of TB diagnosis results for consecutive migrants tested between January I, 1997 and December 31, 2006 were analyzed. We assessed the amplitude (*α*) of the sinusoidal oscillation and the time at which maximum (*θ*°) TB cases were detected using Edwards' test. The adequacy of the hypothesized sinusoidal curve was assessed by *χ*^2 ^goodness-of-fit test.

**Results:**

During the 10 year study period, the proportion (per 100,000) of pulmonary TB cases among the migrants was 198 (4608/2328582), (95% confidence interval: 192 – 204). The adjusted mean monthly number of pulmonary TB cases was 384. Based on the observed seasonal pattern in the data, the maximum number of TB cases was expected during the last week of April (*θ*° = 112°; *P *< 0.001). The amplitude (± se) (*α *= 0.204 ± 0.04) of simple harmonic curve showed 20.4% difference from the mean to maximum TB cases. The peak to low ratio of adjusted number of TB cases was 1.51 (95% CI: 1.39 – 1.65). The *χ*^2 ^goodness-of-test revealed that there was no significant (*P *> 0.1) departure of observed frequencies from the fitted simple harmonic curve. Seasonal component explained 55% of the total variation in the proportions of TB cases (100,000) among the migrants.

**Conclusion:**

This regularity of peak seasonality in TB case detection may prove useful to institute measures that warrant a better attendance of migrants. Public health authorities may consider re-allocation of resources in the period of peak seasonality to minimize the risk of *Mycobacterium tuberculosis *infection to close contacts in this and comparable settings in the region having similar influx of immigrants from high TB burden countries. Epidemiological surveillance for the TB risk in the migrants in subsequent years and required chemotherapy of detected cases may contribute in global efforts to control this public health menace.

## Background

The contribution of immigrants to the changing rate of pulmonary tuberculosis (TB) has been observed in many regions of the world, such as Europe [1], USA [2], Canada [3], Australia [4], and Scandinavian countries [5]. Currently, the proportion of immigrants among prevalent TB cases exceeds 50% in several European countries [6], and in United States [7]. In Kuwait, TB incidence showed a decline from 1965 to 1989. However, from 1989 to 1999, there was a steady increase in the TB incidence among both nationals and non-nationals suggestive of *Mycobacterium tuberculosis *transmission from non-nationals to nationals since a large proportion of migrants from South Asian and South-East Asian countries live and work in Kuwaiti homes as domestic workers [8]. This reversal of the observed TB trend in Kuwait may be explained in part by the disruption of TB control program following first Gulf War in 1990 [8], and by the concomitant increase in the proportion of migrants from high TB burden countries [9]. The epidemiologic importance of migration from high TB incidence to low TB incidence countries has been recognized for several years; the main countermeasure has been the implementation of screening programs for immigrants at the time of arrival, and providing required chemotherapy [10,11].

Trend analysis of TB incidence may help to identify its risk factors and target interventions to prevent it. But it is also important to identify possible seasonal pattern in the disease incidence, the knowledge of which may be used; i. to predict the future magnitude of the health problem; ii. to develop an effective public health program, and iii. to set objectives and utilize available resources more effectively [12]. TB is not widely known to have any seasonal patterns, but few studies have shown variable periods of peak seasonality in TB incidence/case notification rates in late winter to early spring in South Africa [13], during summer in UK [14] and Hong Kong [15], during summer and autumn in Spain [12] and Japan [16]. Relatively recently, it was demonstrated that TB diagnosis peaked between April and June and reached a nadir between October and December in northern India and magnitude of seasonal variation had significant positive correlation with TB case rates [17]. However, to our knowledge, no studies so far have described TB seasonality in Middle East. In Kuwait; there is evidence of seasonal variation in respiratory disease related morbidity and mortality which peaks during winter [18]. Nonetheless, no published data on any seasonal pattern in positive TB diagnosis in Kuwaiti population specifically among migrants from high TB burden countries are available. Therefore, this study examined possible seasonal variation in pulmonary TB cases detected among migrant workers entered Kuwait between January 1, 1997 and December 31, 2006.

## Methods

### The Data

Monthly aggregates of results for TB diagnosis among consecutive migrant workers entered in Kuwait between January 1, 1997 and December 31, 2006 were available for this study. These immigrant workers predominantly came from India (31%), Bangladesh (14%), Sri-Lanka (14%), Egypt (12%), Indonesia (9%), Philippine (5%), Pakistan (5%) and 10% from other countries including those from African counties such as Tanzania, Mali, Gambia, Sudan [19,20]. Routine consensual medical examination procedures were conducted on these workers upon their arrival by the Ports & Borders Health Division of Ministry of Health, Kuwait. For the diagnosis of TB, migrants were screened by the serial application of various tests. For each migrant chest radiograph was taken. In the presence of any suspicious lesion in the lungs, confirmatory TB diagnosis was made by sputum smear examination for acid fast bacilli (AFB) using Ziehl Neelsen technique and bacterial culture. Subsequently, migrant worker was classified as a TB case if sputum smear and/or bacterial culture was positive for AFB [21].

### Statistical methods

**Figure 1:**
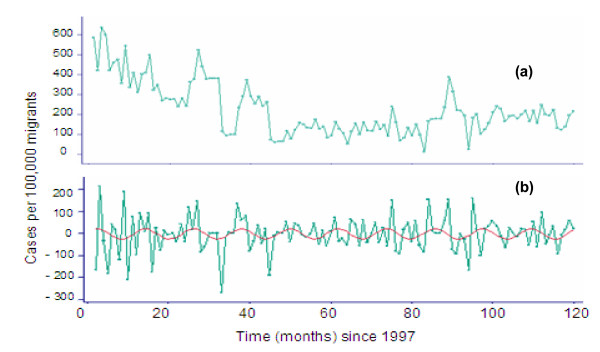
Pulmonary tuberculosis positive (per 100,000/month) migrant workers entering Kuwait, 1997–2006; (a) raw data; (b) de-trended data.

The monthly aggregates of daily number of migrants tested and daily number of TB cases detected were used to generate the monthly series of proportions of TB cases (per 100,000) over a period of 120 months from January 1, 1997 to December 31, 2006. These monthly proportions (per 100,000) of TB cases among migrants were used for all further analyses unless stated otherwise. We computed un-adjusted month-specific (within 12 month period) proportion (95% confidence interval: (CI)) as well as adjusted overall mean monthly proportion (95% CI) of TB cases (per 100,000) for the entire study period. After plotting the raw data (Figure 1a), the evident temporal trends were further explored using smoothing techniques. As the interest of this study was to establish seasonal pattern, a circannual (12 month) cycle was assumed. Therefore, to highlight any potential seasonal effect and to remove any potential trend component, the data were differenced at lag 1 (Figure 1b). The profile of differenced data on proportion of TB cases (per 100,000) suggested that variation might be seasonal in pattern discernable as a sine function with one maximum level and one minimum level per year (Figure 1b).

### Seasonality analysis

As noted above, the profile of the observed month-specific proportions (per 100,000) of TB cases among migrants aggregated over 10-year study period suggested a seasonal pattern discernable as a sinusoidal curve with a period of one year. The significance of unimodal cyclic pattern in the proportion of TB cases was tested by Walter and Elwood's method [22], which takes into account variations in population at-risk. This procedure yields a statistic d = distance from centre of the gravity to the true geometric centre of the circle (*i.e*. the magnitude of the unimodal seasonal trend), which on null hypothesis is distributed as *χ*^2 ^with 2 degrees of freedom. Subsequent to the verification of unimodal nature of the yearly pattern, the seasonality in the data was modelled using a simple sinusoidal harmonic model [23]. Specifically, this model assumes that expected monthly frequencies (*y*_*i*_) are proportional to the simple harmonic curve i.e. *y*_*i *_= *μ *(1 + *α *sin (*θ*_*i *_- θ*)), *where y*_*i *_= expected number of TB cases (per 100,000) for months, *i *= 1, 2,..., 12 (*where *1 = January, 2 = February, etc); *μ *is the mean number of TB cases (per 100,000) detected over the 12-month cycle; *θ*_*i *_is the phase angle representing *i*th month; θ* is the phase angle indicating the month of the maximum frequency of TB cases; *α *is the amplitude (> 0) of the seasonal effect, interpreted as the proportional increase over the mean during θ*. A test of the null hypothesis that *α *= 0 was performed [23]. The adequacy of the description of the data by the hypothesized simple harmonic curve was evaluated by a *χ*^2^goodness-of-fit test. To evaluate the consistency of the seasonal pattern across the entire study period, we split the data into two halves based on two sub-periods (i.e. 1997 to 2001 and 2003 to 2006) and seasonality parameters were re-estimated separately for each of sub-periods as was done for whole data set.

We also decomposed the total sample variance of the distribution of monthly TB cases (per 100,000) into seasonal, non-seasonal and random components [24].

## Results

**Figure 2:**
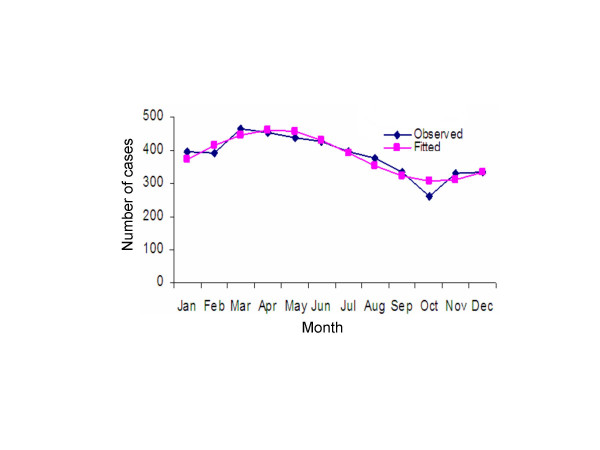
Observed and predicted (Edwards' model) seasonal distribution of pulmonary tuberculosis positive migrants entering Kuwait: 1997–2006.

The un-adjusted observed month-specific proportion of TB cases (per 100,000) among migrants was highest in March (243; 95% CI: 222 – 266) and lowest in October (136; 95% CI: 121 – 152) (Table 1). During the 10-year study period, the un-adjusted mean monthly proportion of TB cases (per 100,000) was 198 (95% CI: 192 – 204). The adjusted mean monthly number of pulmonary TB cases among these migrants was 384. A test of null hypothesis of no seasonal effect (i.e. *α *= 0) was rejected (*P *< 0.001). The Edwards' model revealed that based on the observed seasonal pattern in the data, the significant peak in number of TB cases was expected during the last week of April (*θ*° = 112°; *P *< 0.001). The amplitude (± se) (*α *= 0.204 ± 0.04) of simple harmonic curve showed 20.4% difference from the mean to maximum TB cases among migrants. The peak to low ratio of adjusted number of TB cases was 1.51 (95% CI: 1.39 – 1.65). The *χ*^2 ^goodness-of-test revealed that there was no significant (*P *> 0.01) departure of adjusted observed frequencies from the fitted simple harmonic curve (Table 2; Figure 2). Analysis of the data for two sub-periods (i.e. 1997 to 2001 and 2002 to 2006) to evaluate the consistency of significant seasonal pattern yielded estimates which were nearly consistent with those noted above for the entire period (data not shown). Decomposition of variance in the proportion of TB cases (per 100,000) among migrants into its components revealed seasonal, non-seasonal and random components as 55%, 32% and 13% respectively.

**Table 1:** Month-specific proportions of pulmonary tuberculosis (TB) positive migrants to Kuwait; January 1, 1997 to December 31, 2006.

Month	Total number tested	Number TB positive*	Number positive per 100,000	95% confidence interval
Jan	178370	368	206	186 – 229
Feb	164297	337	205	184 – 228
Mar	185644	451	243	222 – 266
Apr	166039	392	236	214 – 261
May	182717	418	229	208 – 252
June	189071	422	223	203 – 246
July	211845	437	206	188 – 227
Aug	206167	406	197	179 – 217
Sept	204198	356	174	157 – 193
Oct	227062	308	136	121 – 152
Nov	210844	362	172	155 – 190
Dec	202324	351	174	156 – 193
				
Total	2328582	4608	198	192 – 204

* Un-adjusted (crude observed) month-specific number of pulmonary TB cases of the number of migrants tested during that month over the study period

Overall monthly adjusted mean (weighted by average monthly number of migrants i.e. N/12) number of pulmonary TB cases was 384.

**Table 2:** The observed* and expected* number of pulmonary tuberculosis positive migrants by month in Kuwait; Jan 1, 1997 to Dec 31, 2006.

	Jan	Feb	Mar	Apr	May	Jun	Jul	Aug	Sep	Oct	Nov	Dec
Observed	396	394	466	453	439	428	396	378	335	260	330	333
Expected	374	414	446	462	456	432	394	354	322	306	312	336

* The month-specific observed number of cases is weighted by the average monthly number of migrants tested (i.e. observed proportion of TB positives in given month × average monthly number of workers tested during the entire study period; i.e. N/12). Subsequently weighted number of observed cases was re-adjusted to have total weighted number of TB cases equal to total number of actual observed cases.

(*e.g*. For January observed weighted number of cases = [(206/100,000) × 194049] = 400.292 ≅ 400 (after rounding down)

For January, re-adjusted observed weighted number of cases = (400/4659) × 4608 = 396

*where; 4659 is the total observed weighted number of cases calculated from January through December by rounding up or down as required and 4608 are actual number of observed cases in the study*..

The expected (calculated for the best simple harmonic curve) number of cases has been adjusted by the number of days in the month *χ*^2 ^= 0.224; df = 11 *P *> 0.1

## Discussion

The findings of this study suggested that there was a spring peak (late April) in TB cases detected among migrants entering Kuwait from high TB burden countries. The estimated amplitude of seasonal variation in the number of TB cases was 20.4% from the annual mean. The observed amplitude of seasonal variation was substantial enough to be explained by monthly variations in the number of TB cases detected among these migrants. In the context of TB diagnosis/case notification rates, few studies have reported variable peaks of seasonality from different parts of the world. For example, in the pre-antibiotic era, TB related mortality was higher in the late winter and early spring than at any other time of the year [25]. Relatively few recent studies have shown a similar seasonal pattern in TB notification rates [13,26,27]. Also, peak seasonality has been reported during summer in UK [14], and Hong Kong [15], during summer and autumn in Spain [12], Japan [16], and April to June in India [17]. Exactly, why TB diagnosis/case notification rates might vary by season is unknown and the specific contributions of a variety of climatic and meteorological seasonal changes are uncertain. However, it has been suggested that winter indoor crowding in poorly ventilated settings could lead to increased *M. tuberculosis *transmission, which then manifests itself 3–6 months later [13]. As noted above, the preclinical period, from exposure to clinical onset, may be of several weeks, which may in part, explain variable seasonal peaks in different geographic regions [12,13,16].

In this study, all of the detected TB cases were adults, and most of the TB cases in adults are considered to be the results of reactivation of latent *M. tuberculosis *infection. In the absence of HIV infection or immunosuppressive therapy, such TB cases resulting from re-activation of latent *M. tuberculosis *infection are attributed to poor nutrition and low socio-economic status. Although the exact mechanism of this re-activation of *M. tuberculosis *infection remains unclear [28], yet cell mediated immunity in *M. tuberculosis *infection seems to play role because of circannual variation in lymphocyte subsets. The seasonal changes in the absolute numbers and ratios of T helper and T suppressor cells could possibly alter cell mediated immunity that controls host response to *M. tuberculosis *infection. Nevertheless, the factors that regulate the seasonal changes in T cell subset numbers or function remain unknown [29]. It has been argued however, that in winter and spring, the viral infections like flu, are more frequent and cause immunological deficiency leading to re-activation of *M. tuberculosis *infection [12]. Furthermore, a probable link between impaired host immunological defence due to vitamin D deficiency and the re-activation of latent *M. tuberculosis *infection has been hypothesized [30-32]. The principal source of vitamin D is ultraviolet radiation from sunlight, and that plasma concentrations of vitamin D have a striking seasonal variation with peak levels after the summer and lowest levels in the spring [31,33]. Also, a significant trend of increasing TB risk with increased vitamin D deficiency due to low frequency of meat or fish consumption among vegetarian Asians has been reported [28]. Since most of the migrants entered in Kuwait during the study period came from South Asia. Therefore, period for the peak seasonality in late April in this study corroborated the findings of reported seasonality in April – June in northern India [17]. Furthermore, the researchers of the aforementioned study have suggested that winter transmission of *M. tuberculosis *due to increased indoor activity and/or vitamin D deficiency leading to re-activation of latent infection may have been the bases for the observed TB seasonality in northern India [17,34]. Perhaps similar biological phenomena may have been associated with significant seasonality in late April among migrants in our study. Also, it may well be that the migrants from India have a very high proportion of TB and arrives mainly in winter months while migrants from Sri Lanka and other low burden countries have a low proportion of TB and arrive mainly in summer. Such workers' recruitment pattern may have potentially contributed in seasonal variation in pulmonary TB in migrants entering Kuwait. However, we did not have season-specific recruitment data nested within the countries of origin of these migrants to support this contention. Nonetheless, exact biological basis for period of peak seasonality during April in this population needs further investigations.

### Limitations of the study

Limitation of this analysis included, firstly, the data deficiency on demographic characteristics of migrants e.g. age and gender. The variation in amplitude of seasonal fluctuation in TB notification rates by age has been reported [15]. As noted above, we did not have data on exact birth dates of migrants to compute age-specific amplitudes of seasonal variation in our study. However, all the migrants were adults aged 18 or more and relatively small variation in seasonal amplitudes for higher age groups was reported [15]. Furthermore, no difference in TB seasonal pattern by gender was observed previously [15,17]. Secondly, we did not have data on various tests results on migrants for comparative presentation other than the final diagnosis for each migrant as TB positive or TB negative which we believe was done with a sufficient level of accuracy. Thirdly, some level of misclassification of TB cases or non-TB migrants might have occurred as intra-observer and inter-observer variations in radiological assessment of TB cases have been reported [35,36]. However, this misclassification presumably was minimal because of serial application of battery of screening and confirmatory tests on our study population. Also, for this task, Kuwait Public Health Authority ensured to employ experienced radiologists who are known to have the highest level of agreement in chest radiograph reading compared to observers of other specialities [35]. Finally, the monthly grouped data precluded a more sensitive day-by-day assessment of TB seasonality in this population.

## Conclusion

Taken together, the results of this and previous studies seem to corroborate the evidence for seasonality in detection of TB cases, which peaks in late April in this migrant population. This regularity of peak seasonality in TB cases may prove useful to institute measures that warrant a better attendance of migrants. Public health authorities may consider re-allocation of resources in the period of peak seasonality to minimize *M. tuberculosis *infection risk to close contacts in this and comparable settings in the region having similar influx of migrants from high TB burden countries. Epidemiological surveillance for the TB risk in the migrants in subsequent years and required chemotherapy of detected cases may contribute in global efforts to control this public health menace.

## Competing interests

The author(s) declare that they have no competing interests.

## Authors' contributions

SA conceived, design, analyzed and interpreted data and drafted the manuscript. HGHM supervised data collection and reviewed the manuscript. Both the authors have read and approved the final manuscript.

## Pre-publication history

The pre-publication history for this paper can be accessed here:


